# TissuePatch™ as a novel synthetic sealant for repair of superficial lung defect: in vitro tests results

**DOI:** 10.1186/1750-1164-6-12

**Published:** 2012-11-19

**Authors:** Ruoyu Zhang, Maximilian Bures, Hans-Klaus Höffler, Norman Zinne, Florian Länger, Theodosios Bisdas, Axel Haverich, Marcus Krüger

**Affiliations:** 1 Department of Cardiac, Thoracic, Transplantation and Vascular Surgery, Hannover Medical School, Carl-Neuberg Str. 1, Hannover, 30625, Germany; 2 Department of Pathology, Hannover Medical School, Carl-Neuberg Str. 1, Hannover, 30625, Germany

**Keywords:** Lung, Pleural air leak, Sealant, In vitro model

## Abstract

**Background:**

Controversies surrounding the efficacy of surgical sealants against alveolar air leaks (AAL) in lung surgery abound in the literature. We sought to test the sealing efficacy of a novel synthetic sealant, TissuePatch™ in an in vitro lung model.

**Methods:**

The lower lobe of freshly excised swine lung (n = 10) was intubated and ventilated. A superficial parenchymal defect (40 × 25 mm) was created, followed by AAL assessment. After sealant application, AAL was assessed again until burst failure occurred. The length of defect was recorded to evaluate the elasticity of the sealant.

**Results:**

Superficial parenchymal defects resulted in AAL increasing disproportionally with ascending maximal inspiratory pressure (Pmax). Multiple linear regression analysis revealed strong correlation between AAL and Pmax, compliance, resistance. After sealant application, AAL was sealed in all ten tests at an inspired tidal volume (TVi) of 400 ml, in nine tests at TVi = 500 ml, in seven at TVi = 600 ml and in five at TVi = 700 ml. The mean burst pressure was 42 ± 9 mBar. Adhesive and cohesive sealant failures were found in six and three tests respectively. The length of defect before sealant failure was 8.9 ± 4.9% larger than that at TVi = 400 ml, demonstrating an adequate elasticity of this sealant film.

**Conclusions:**

TissuePatch™ may be a reliable sealant for alternative or adjunctive treatment for repair of superficial parenchymal defects in lung surgery. The clinical benefits of this sealant should be confirmed by prospective, randomised controlled clinical trials.

**Abstrakt:**

**Hintergrund**

Die Wirksamkeit von chirurgischen Klebstoffen zur Prävention von alveolo-pleuralem Luftleck (APL) ist trotz zunehmenden klinischen Anwendungen in Lungenchirurgie immer noch kontrovers diskutiert. Wir evaluierten die Abdichtungswirksamkeit von einem neuartigen synthetischen Kleber, TissuePatch™ mittels eines in vitro Lungenmodels.

**Methode:**

Der Unterlappen von frisch entnommenen Schweinlungen (n = 10) wurde intubiert und beatmet. Eine pleurale Läsion (40 × 25 mm) wurde erstellt und APL mit steigendem inspiratorischem Tidalvolumen (TVi) untersucht. Nach Applikation von TissuePatch™ wurde APL auf die gleiche Weise gemessen bis zur Auftritt von Kleberbruch. Zur Untersuchung der Elastizität des Klebers wurde die Länge der pleuralen Läsion gemessen.

**Ergebnis:**

Pleurale Läsion führte bei aufsteigendem maximalem inspiratorischem Druck (Pmax) zu überproportionalem Anstieg von APL. Multiple lineare Regressionsanalyse ergab eine starke Korrelation zwischen APL und Pmax, Lungencompliance sowie Widerstand. Nach der Applikation von Klebstoff wurde APL bei TVi = 400 ml in allen zehn Testen versiegelt, bei TVi = 500 ml in neun Testen, bei TVi = 600 ml in sieben und bei TVi = 700 ml in fünf Testen. Der mittlere Pmax, der zu Kleberbruch führte, betrug 42 ± 9 mBar. Bei den Versuchen wurden adhäsiver und kohäsiver Kleberbruch in jeweils sechs und drei Testen gefunden. Die Länge der pleuralen Läsion vor dem Kleberbruch war 8,9 ± 4,9% größer als die bei TVi = 400 ml.

**Schlussfolgerung:**

Unsere Versuche zeigten eine zuverlässige Versiegelung von TissuePatch™ unter mechanischer Ventilation. Die klinische Nützlichkeit vom Kleber als unterstützende Maßnahme zur Prävention von alveolo-pleuralem Luftleck in Lungenchirurgie sollte durch prospektive, randomisierte kontrollierte klinische Studien bestätigt werden.

## Background

Alveolar air leaks (AAL) resulting from superficial lung parenchymal defects are a common intra- and post-operative complication in lung surgery, especially in pleural decortication, dissection of firm pleural adhesion and division of incomplete fissures [1]. They result in delayed chest tube removal, prolonged hospital stay and marked patient discomfort as well as higher health care costs [1,2]. In the last decade, various surgical sealants have been developed and become progressively widespread to prevent AAL in lung surgery [1,3]. As adjuncts to conventional closure techniques, application of surgical sealants has been reported to be able to reduce mean duration of AAL, duration to chest tube removal [4-6] and the length of hospital stay [4,6,7], as well as incidence of postoperative empyema [8,9]. However, according to the current Cochrane review and meta-analysis of Malapert et al., randomized controlled clinical trials assessing surgical sealants in treating AAL demonstrated inconsistent results, raising a question regarding their efficacy [1,10].

**Figure 1:**
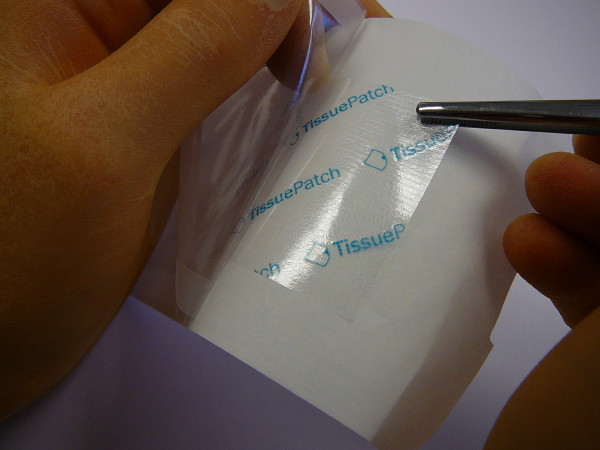
**TissuePatch**^**TM**^**synthetic sealant film.**

TissuePatch™ (TP, Tissuemed Ltd, Leeds, UK) is a novel synthetic sealant, which is self-adhesive and absorbable (Figure 1). It consists of TissueBond (a bioadhesive polymer) and polylactide-co-glycolide (PLGA). The active chemistry of TissueBond forms cross-links at proteins that are present at the site of application, while PLGA functions as a barrier and structural layer and is largely absorbed within 70 days. Since the introduction in 2007, the original product, TissuePatch3™ has been used as an adjunctive treatment in control and prevention of air, blood or fluid leakage in various surgical procedures. Recently, TP as the new generation product is available. In the present study, we sought to examine the sealing efficacy of TP in treating AAL by means of an in vitro lung model.

## Methods

### Experimental protocol

**Figure 2:**
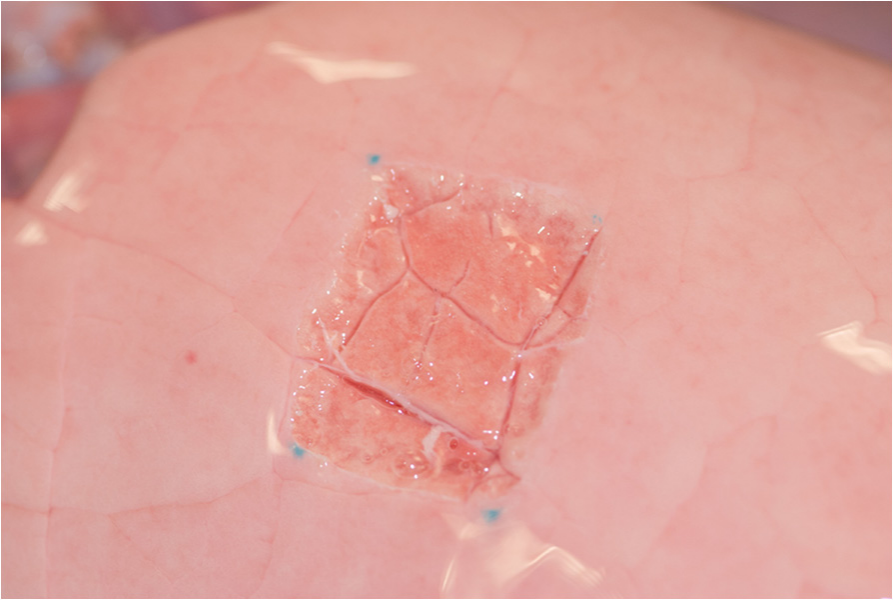
A superficial lung defect was created in a previously marked area of 40 × 25 mm on the inflated lower lobe.

Lungs were freshly excised from German Landrace pigs (around 80 kg) without preference in gender, which were euthanized in a local slaughterhouse. Within two hours after harvest, the lungs were dissected along the trachea until the tracheal bifurcation was reached. The lower lobe was selectively intubated due to the greater facility compared to upper lobe. It was then inflated and immersed in warm water to ensure its impermeability. After being connected to the ventilation machine (Evita, Dräger, Lübeck, Germany), the lobe was ventilated in a volume-controlled mode with positive end-expiratory pressure (PEEP) of 5 mBar, I:E ratio of 1:2 and ventilation frequency of 12/min. The lobe was fully inflated when inspired tidal volume (TVi) ≥ 400 ml, while over-inflation was observed when TVi ≥ 800 ml. A superficial parenchymal lesion (40 × 25 mm) was created on the inflated lobe (Figure 2). Surgical knots were then tied on the cranial and caudal edge of the lesion and served as markers for the measurement of lesion length. Inspiratory volume was increased in steps, recording the respiratory parameters including the expiratory tidal volume (TVe), Pmax, mean inspiratory pressure (Pmean), plateau inspiratory pressure (Pplat), resistance and compliance for each subsequent step until a Pmax of 40 mBar was reached. AAL was calculated as the difference between TVi and TVe. Sealing was considered, if AAL was ≤ 20 ml.

Thereafter, the sealant was applied according to the usage guide, respecting a safety margin of 1 cm to all sides. Mild pressure was applied for a period of 60 second to the sealant film to ensure full adhesion. The lobe was then ventilated again with TVi rising slowly from 100 mL. Commencing at 400 ml TVi, the same respiratory parameters were measured again. In the moment that sealant failure occurred, Pmax was recorded as the burst pressure. The burst failure was categorized into adhesive or cohesive failure. Adhesive failure was considered, if the sealant failure occurred at the interface between the sealant and the parenchymal defect. Cohesive failure was defined as the failure within the sealant.

Finally, the lung specimens containing the parenchymal lesion along with the attached sealant were resected and fixed with 10% formalin. The specimens were further embedded in paraffin, and processed to obtain sections for haematoxylin-eosin staining.

### Statistical analysis

The normality of variables was tested by the Kolmogorov-Smirnov one-sample test. Descriptive statistics are presented as mean ± standard deviation in case of normal distribution. Categorical variables are expressed as percentages. Continuous data was compared using student *t*-test. Multiple linear regression analysis was used to determine the ventilation parameters correlated with AAL. Statistical significance was assumed if *p* < 0.05. All statistical evaluation was performed using SPSS (version 16.0 for Windows; SPSS, Inc., Chicago, IL).

## Results

After establishing the experimental procedures, four pilot tests were performed. The results from pilot tests were not included in the data set or statistical analysis. Subsequently, a total of ten consecutive tests were undertaken. One TP film was applied on one lower lobe in every single test. The recorded ventilation parameters and measured AAL after creation of a superficial parenchymal lesion are listed in Table 1. With ascending inspiratory pressure, AAL increased disproportionally. Multiple linear regression analysis revealed strong correlation between AAL and Pmax, compliance and resistance (p < 0.001, p = 0.01 and p = 0.002, respectively).

**Table 1:** Air leak assessment before sealant application

**TVi (ml)**	**Air leak (ml)**	**Pmax (mBar)**	**Pplat (mBar)**	**Pmean (mBar)**	**Compliance (ml/mBar)**	**Resistance (mBar/l/s)**
400	105 ± 51	24 ± 4	14 ± 1	8 ± 1	34 ± 7	4 ± 1
500	153 ± 64	29 ± 4	16 ± 2	9 ± 1	33 ± 6	5 ± 1
600	214 ± 75	34 ± 5	17 ± 2	10 ± 1	32 ± 5	5 ± 1
700	270 ± 87	38 ± 4	19 ± 2	11 ± 1	32 ± 5	5 ± 1
800	330 ± 92	39 ± 1	21 ± 2	11 ± 1	30 ± 5	6 ± 1
900	380 ± 110	41 ± 3	25 ± 3	12 ± 0	27 ± 4	7 ± 1
1000	438 ± 119	39 ± 1	32 ± 3	13 ± 0	20 ± 5	9 ± 1

Pmax = maximal inspiratory pressure.

Pmean = mean inspiratory pressure.

Pplat = plateau inspiratory pressure.

TVi = inspired tidal volume.

**Figure 3:**
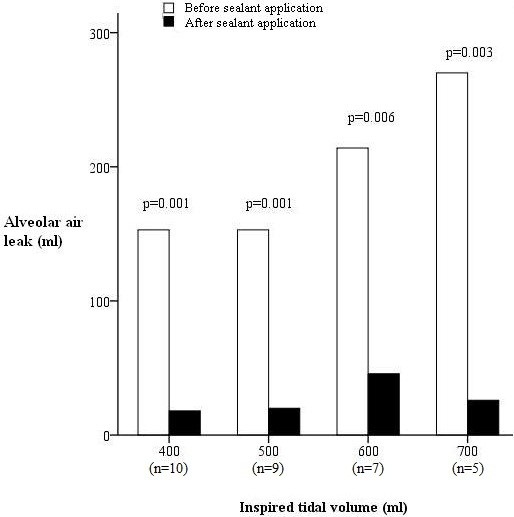
AAL was significantly reduced after application of sealant film at moderate ventilation.

After application of the sealant film, AAL was sealed in all ten tests at TVi of 400 ml, in nine tests at TVi of 500 ml, in seven tests at TVi of 600 ml and in five tests at TVi of 700 ml (Figure 3). As TVi increased ≥ 700 ml, sealing effect was still achieved in one test at TVi of 800 ml and Pmax of 42 mBar (AAL = 0 ml) and in another at TVi of 900 ml and Pmax of 50 mBar (AAL = 20 ml). One test demonstrated complete sealing of AAL even at TVi >1000 ml and Pmax > 60 mBar. The mean burst pressure was 42 ± 9 mBar. Adhesive and cohesive sealant failures were found in six and three tests respectively. The inter-suture distance before burst failure was 8.9 ± 4.9% larger than that at TVi of 400 ml, indicating an adequate elasticity of this sealant film.

**Figure 4:**
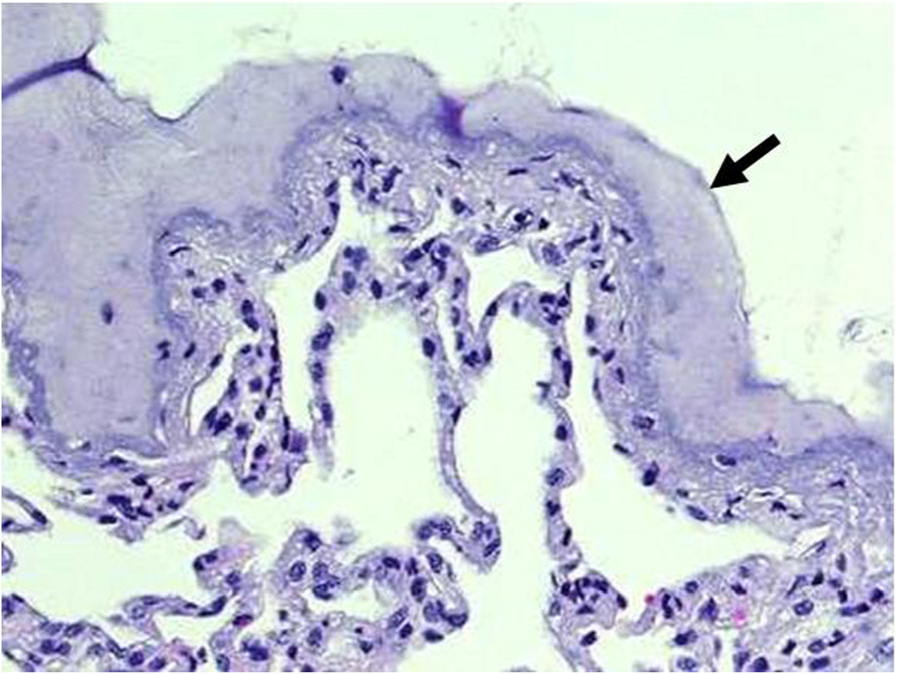
Photomicrograph of histological sections showed the sealant layer (arrow) attaching densely the underlying parenchymal lesion.

Haematoxylin-eosin staining of the lung specimen sealed by TP showed the layer of adhesive polymer attaching densely the underlying parenchymal lesion (Figure 4). The PLGA film was detached during processing of the samples and was not visible.

## Discussion

As adjuncts to suturing or stapling, various surgical sealants have been developed and progressively practised in treating AAL in the last decade. According to a survey of European Association for Cardio-Thoracic Surgery and European Society of Thoracic Surgeons, more than 60% of surgeons used sealants in the practice routinely or when indicated [3]. However, the sealant use was mostly not based on clinical evidence. This is believed to be due to inconsistent data of the published clinical trials assessing the sealing efficacy of surgical sealants. In the current Cochrane review including a total of 16 randomized controlled clinical trials investigating the sealants for preventing air leaks after pulmonary resections, only three trials demonstrated a significant reduction in the length of hospital stay in the treatment group [1]. Post-operative chest tube time was significantly reduced in treatment group only in three trails. Significant difference in duration of air leaks was only found in six out of twelve clinical trails. Aside from some design flaws of these studies, length of hospital stay and duration of chest tube are inadequate primary endpoints to examine the sealant’s efficacy, as they are determined not only by AAL, but also by various other factors including relevant co-morbidities, inadequate post-operative pain control, prolonged fluid output. In contrast, the in vitro lung model in the present study provides a reliable means to assess AAL quantitatively and is able to test the burst pressure of sealant. Furthermore, the elasticity of sealant has also been evaluated. More clinical relevance could be found to compare the sealing efficacy of different sealants in treating AAL with this in vitro model.

TP is a novel, fully synthetic, self-adhesive sealant patch, which is biodegradable. It consists of TissueBond as an adhesive polymer, PLGA and methylene blue. Since the first launch in 2007, the original product, TissuePatch3^TM^ has been progressively widespread as an adjunct to seal leakage of air, chylous fluid and blood in lung surgery, thyroidectomy, major neck surgery as well as in the prevention of cerebrospinal fluid leaks following neurosurgery. In the present study, we demonstrated the strong sealing efficacy of TP as the new generation product under mechanical ventilation. The mean burst pressure exceeded the upper limit of the inspiratory pressure, which is typical in most clinical settings (Pmax ≤ 40 mBar). In the present experiment, certain variation in the size of ventilated lower lobes could not be totally avoided. However, we did not observe remarkable difference in this regard during the tests. It could be explained that the lungs were harvested from the pigs in almost the same weight (around 80 kg). In all tests, the lower lobe was fully inflated when TVi was 400 ml or higher, indicating the absence of significant difference in the size of lower lobes.

In a recent in vitro experiment comparing the sealing efficacy of six different sealants including TissuePatchDural^TM^ (TPD, Tissuemed Ltd, Leeds, UK) similar to TissuePatch^TM^, Pedersen et al. fixed harvested porcine lungs in a Plexiglas chamber filled with isotonic saline [11]. After sealant application on deflated lungs, the lungs were ventilated with incremental peak airway pressure and air leaks were assessed by visual inspection of air bubbles in submersion tests. Their results demonstrated a very low median burst pressure of TPD (25 mBar). The sealant never withstood peak pressures higher than 30 mBar. Compared to the in vitro model of the present experiment, certain factors may have biased their study to the disadvantage of TPD. Firstly, the TPD film was applied to deflated lungs, whereas the manufacturer recommends that the lung should be ¾ inflated during application. The subsequent lung re-inflation and stretching may have deteriorated the bonding between the adhesive polymer and lung surface due to break of the cross-links. Furthermore, the time from lung harvest to experimentation averaged as long as 24 hours. The resulted protein degradation may also have impaired the cross-linking of polymer at lung tissue, resulting in weaker sealing efficacy. Additionally, air leakage assessment was performed via inspection of air bubbles, and observer blinding was not possible due to the obvious differences in product appearance in their experiment.

Our results are comparable to the previously published data of fibrin sealant patches in in vivo animal experiments. In experimental studies with beagles, the working group of Dr. Kawamura and Dr. Gika made defects on the lung surface (5 × 10 mm and 5 × 20 mm, respectively) and evaluated the sealing effect of fibrin glue against air leakage with different methods of application. In the group using collagen fleece, coated with fibrinogen and thrombin (TachoComb®, ZLB Behring Co., King of Prussia, PA, USA), the mean seal breaking pressure was as high as 36 ± 6 mBar and slightly over 40 mBar, respectively [12,13].

TP is a ready to use sealant, which does not require any preparation before application. It bonds to surgical site in 60 seconds. Compared to TP, fibrin patches such as TachoComb® or TachoSil® (Takeda, Zurich, Switzerland) need to be moistened before application, with pressure required for 3 to 5 minutes after application according to the usage guide. Furthermore, fibrin sealants are typically derived from human or bovine blood plasma, exposing patients to the potential risk of transmission of blood-borne diseases [5,12]. Kamamura et al. reported infection of human parvovirus B19 in more than 20% of patients following use of fibrin sealant during lung resection [14]. In this respect, application of TP as a fully synthetic, biodegradable material completely eliminates this risk. As a further advantage, TP can be delivered thoracoscopically by means of a dedicated delivery system. Nevertheless, it is of utmost importance in confirming the clinical benefits of TP by means of prospective, randomised controlled clinical trials.

## Conclusions

Based on our results, the use of TissuePatch™ in lung surgery as a treatment for the adjunctive repair of superficial parenchymal defects, even under aggressive mechanical ventilation, is recommended. In vitro evaluation also demonstrated adequate elasticity of this sealant film. The clinical and financial benefits of this sealant film should be confirmed by prospective, randomised controlled clinical trials.

## Abbreviations

AAL: Alveolar air leak; TVi: Inspired tidal volume; Pmax: Maximal inspiratory pressure; PLGA: Polylactide-co-glycolide; PEEP: Positive end-expiratory pressure; TVe: Expiratory tidal volume; Pmean: Mean inspiratory pressure; Pplat: Plateau inspiratory pressure.

## Competing interests

The surgical sealants tested in the present study were provided by the manufacturer (Tissuemed Ltd, Leeds, UK) for testing purposes. All authors have no financial or other interests regarding the submitted manuscript.

## Authors’ contributions

RZ carried out the conception and design of the study, in vitro tests, acquisition of data, analysis and interpretation of the data, statistical analysis as well as drafting of the manuscript. MB participated in the in vitro tests, analysis and interpretation of the data, drafting of the manuscript and critical revision of the manuscript. KH and NZ participated in the acquisition of data. FL participated in histological examination. TB and AH participated in the analysis and interpretation of the data, critical revision of the manuscript and supervision of the study. MK participated in the conception and design of the study, acquisition and interpretation of the data, drafting and critical revision of the manuscript. All authors read and approved the final manuscript.
